# Open Lift, Drill, Fill, and Fix (LDFF) for Chronic Osteochondral Lesions of the Talus: Favorable 2-Year Clinical Outcomes

**DOI:** 10.1177/23259671251356700

**Published:** 2025-08-08

**Authors:** Q.G.H. Rikken, Jari Dahmen, Kaj T.A. Lambers, Kaj S. Emanuel, Sjoerd A.S. Stufkens, Gino M.M.J. Kerkhoffs, Amsterdam Ankle Cartilage Team, J. Nienke Altink, Christiaan J.A. van Bergen, Peter A.J. de Leeuw, Rover Krips, Mikel L. Reilingh

**Affiliations:** Department of Orthopedic Surgery and Sports Medicine, Amsterdam UMC, Location AMC, University of Amsterdam, Amsterdam, the Netherlands; Amsterdam Movement Sciences, Programs Sports and Musculoskeletal Health, Amsterdam, the Netherlands; Academic Center for Evidence-based Sports medicine, Amsterdam UMC, Amsterdam, the Netherlands; Amsterdam Collaboration on Health & Safety in Sports, International Olympic Committee Research Center, Amsterdam UMC, Amsterdam, the Netherlands; Department of Orthopedic Surgery and Sports Medicine, Amsterdam UMC, Location AMC, University of Amsterdam, Amsterdam, the Netherlands; Amsterdam Movement Sciences, Programs Sports and Musculoskeletal Health, Amsterdam, the Netherlands; Academic Center for Evidence-based Sports medicine, Amsterdam UMC, Amsterdam, the Netherlands; Amsterdam Collaboration on Health & Safety in Sports, International Olympic Committee Research Center, Amsterdam UMC, Amsterdam, the Netherlands; Amsterdam Movement Sciences, Programs Sports and Musculoskeletal Health, Amsterdam, the Netherlands; Academic Center for Evidence-based Sports medicine, Amsterdam UMC, Amsterdam, the Netherlands; Amsterdam Collaboration on Health & Safety in Sports, International Olympic Committee Research Center, Amsterdam UMC, Amsterdam, the Netherlands; Reinier Haga Orthopaedic Centre, Zoetermeer, the Netherlands; Amsterdam Movement Sciences, Programs Sports and Musculoskeletal Health, Amsterdam, the Netherlands; Academic Center for Evidence-based Sports medicine, Amsterdam UMC, Amsterdam, the Netherlands; Amsterdam Collaboration on Health & Safety in Sports, International Olympic Committee Research Center, Amsterdam UMC, Amsterdam, the Netherlands; Department of Orthopedic Surgery and Sports Medicine, Amsterdam UMC, Location AMC, University of Amsterdam, Amsterdam, the Netherlands; Amsterdam Movement Sciences, Programs Sports and Musculoskeletal Health, Amsterdam, the Netherlands; Academic Center for Evidence-based Sports medicine, Amsterdam UMC, Amsterdam, the Netherlands; Amsterdam Collaboration on Health & Safety in Sports, International Olympic Committee Research Center, Amsterdam UMC, Amsterdam, the Netherlands; Department of Orthopedic Surgery and Sports Medicine, Amsterdam UMC, Location AMC, University of Amsterdam, Amsterdam, the Netherlands; Amsterdam Movement Sciences, Programs Sports and Musculoskeletal Health, Amsterdam, the Netherlands; Academic Center for Evidence-based Sports medicine, Amsterdam UMC, Amsterdam, the Netherlands; Amsterdam Collaboration on Health & Safety in Sports, International Olympic Committee Research Center, Amsterdam UMC, Amsterdam, the Netherlands; Department of Orthopaedic Surgery and Sports Medicine, Amsterdam UMC, Location AMC, University of Amsterdam, Amsterdam, the Netherlands; Amsterdam Movement Sciences, Programs Sports and Musculoskeletal Health, Amsterdam, the Netherlands; Academic Center for Evidence-based Sports medicine, Amsterdam UMC, Amsterdam, the Netherlands; Amphia Hospital, Breda, the Netherlands; Flevoziekenhuis, Almere, the Netherlands; Flevoziekenhuis, Almere, the Netherlands; St. Antonius Hospital, Utrecht, the Netherlands; Investigation performed at Department of Orthopaedic Surgery and Sports Medicine, Amsterdam UMC, Amsterdam, the Netherlands

**Keywords:** lift, drill, fill and fix, LDFF, internal fixation, osteochondral lesion of the talus (OLT)

## Abstract

**Background:**

In the presence of an osteochondral fragment with sufficient subchondral bone thickness, fixation is considered to be an effective treatment method for osteochondral lesions of the talus (OLT). One such fixation technique is the lift-drill-fill-fix (LDFF) procedure, which has shown reliable long-term results in an arthroscopic approach; however, the outcomes in cases treated through an open approach are unknown.

**Purpose:**

To assess the 2-year outcomes after open LDFF for chronic OLT.

**Study Design:**

Case series; Level of evidence, 4.

**Methods:**

A total of 34 patients who underwent an open LDFF procedure for chronic (>6 weeks) OLT were prospectively followed for 2 years. The primary outcome concerned the comparison in numeric rating scale of pain (0 = no pain; 10 = most severe pain) during walking between the preoperative score to the 2-year postoperative follow-up score. The association of baseline factors with the change in the primary outcome between baseline and 2-year follow-up was assessed. Secondary patient-reported outcome measures (PROMs) were the Foot and Ankle Outcome Score and 36-Item Short Form Health Survey (SF-36). The fragment union rate on 1-year follow-up computed tomography scans and the influence of possible baseline factors on union were assessed. Adverse events, including revision surgery and complications, were assessed and documented.

**Results:**

The primary outcome significantly improved from a median of 6 (IQR, 4-7) out of 10 preoperatively to 1 (IQR, 0-3) out of 10 at final follow-up, *P* < .01. There was no association between baseline factors (sex, age, body mass index (BMI), smoking status, lesion size, and location) and change in primary outcome between baseline and 2-year follow-up. All other PROMs significantly improved, except for the SF-36 Mental Component Summary. The fragment union rate was 91% [95% CI, 76-98]. BMI of ≥30 kg/m^2^ was significantly associated with fragment nonunion (odds ratio, 1.39; 95% CI, 1.04-1.84; *P* = .02). Three patients underwent revision surgery, while 2 complications (1 case of delayed superficial wound healing and 1 case of complex regional pain syndrome) were observed.

**Conclusion:**

Open LDFF resulted in favorable PROs for chronic OLT up to 2-year follow-up. The procedure achieved a 91% fragment union rate, while patients with obesity showed a higher risk of fragment nonunion.

Osteochondral lesions of the talus (OLT) concern a disruption of the articular cartilage and the underlying subchondral bone. Such lesions may cause complaints of pain during activities such as sports.^24^ The current treatment of symptomatic OLT is based on a patient- and lesion-specific treatment algorithm and consists of non-operative and operative treatment options.^24^ Important factors for the treatment decision are the lesion morphology and size.^20,24^

A distinct morphological OLT type includes the presence of an osteochondral fragment.^27^ In such lesions with an osteochondral fragment, the articular cartilage can be intact although the subchondral bone is compromised.^14^ Fixation techniques were developed to exploit this opportunity, as they aim to retain the native hyaline cartilage, stabilize the osteochondral fragment through direct fixation, and restore the joint congruency.^21,27^ A recent systematic review found that fixation techniques for OLT show good clinical results, with high union rates and low revision rates.^23^

One such fixation technique is the lift-drill-fill-fix (LDFF) technique. This technique is tailored to chronic lesions, which can be seen as an intra-articular nonunion. They require subchondral bone repair through drilling of the lesion site, autologous bone grafting, and subsequent fixation (compression).^8,11,26^ The LDFF technique was initially introduced as an arthroscopic technique that showed good midterm results that were retained up to long-term follow-up.^11,25^ A limitation of the arthroscopic technique is the accessibility to centrally and posteriorly located OLT, especially on the medial side, or lesions too large to fix arthroscopically. A difficult-to-reach or inaccessible lesion may lead to inappropriate screw placement, periscrew cysts, and insufficient stability after fixation.^8,16^ Moreover, it is known that the majority of OLT are located on the central or posterior medial talar dome.^31^ Therefore, an open version of the LDFF technique was developed that showed promising clinical results in a sample of 13 patients.^26^ The midterm outcomes of the open LDFF technique remain to be elucidated, however.

As such, the primary aim of the present study was to assess the 2-year prospective patient-reported clinical outcomes after open LDFF for chronic OLT with an osteochondral fragment. Secondarily, this study evaluated the radiological outcomes and adverse events. We hypothesized that the open LDFF technique leads to an improvement in 2-year patient-reported outcomes with a high union rate of the fixed fragment.

## Methods

### Study Design

This study is a prospective, single-center, case series with a 2-year follow-up. The study was approved by the local medical ethics committee at Amsterdam UMC, location AMC, and was performed per the current ethical standards (Declaration of Helsinki).

### Patient Selection

All prospectively followed patients who had a symptomatic chronic OLT with osteochondral fragment, confirmed per computed tomography (CT) scan, and who were treated with LDFF through an open approach from July 2015 to July 2022 were assessed for the inclusion and exclusion criteria (Table 1). The indication for an open medial LDFF procedure was a symptomatic chronic osteochondral fragment of the medial or central talus, eligible for in situ fixation (≥10-mm diameter and ≥3-mm thick as per CT scan), that failed 3 to 6 months of non-operative treatment.^26^ The contraindications were previously described.^26^ Laterally located OLT treated by LDFF adhered to the same indication criteria, except for the omitted surgical contraindication of open physes. The decision for an open approach was made on surgeon’s discretion and based on preoperative planning (ankle range of motion and evaluation of the lesion [size and location] and bony structures on CT scan) to establish if an open approach was necessary for sufficient access to the lesion for successful fixation. The study site concerned a tertiary academic referral hospital that is recognized as an expert center in the diagnosis and treatment of OLT.

**Table 1:** Inclusion and Exclusion Criteria*^*a*^*

Inclusion Criteria	Exclusion Criteria
Symptomatic chronic osteochondral fragment of the talus, eligible for in situ fixation,^26^ and failing 3-6 months of non-operative treatment	Patients presenting with a symptomatic acute lesion (<6 weeks after clearly identifiable trauma) requiring immediate fixation Concomitant ankle or hindfoot fracture at the time of the LDFF surgery Severe developmental disorder of the foot and ankle Patients unwilling or unable to participate

a LDFF, lift, drill, fill, and fix.

### Surgical Technique

The surgical technique for the open LDFF procedure was previously described in detail for medial or centrally located OLT.^26^ In summary, the joint was accessed through either a medial arthrotomy or a distal tibial osteotomy. In case of a lateral OLT, an anterolateral arthrotomy was performed where it was necessary to release the anterior talofibular ligament or perform an osteotomy of the distal fibula to achieve good visualization of the affected part of the talus, as previously described.^4^ After identification of the osteochondral fragment, the articular cartilage around the fragment was partially incised to allow lifting of the fragment (lift). The subchondral bone was debrided and drilled for marrow stimulation (drill), whereafter the autologous bone graft from the distal tibia (osteotomy) or iliac crest was used to fill the site (fill). Hereafter, the fragment was anatomically fixed and compression and rotational stability were provided by preferably 2 screws (and/or darts). In the initial experience, the senior authors (S.A.S.S. and G.M.M.J.K.) utilized metallic screws, but based on clinical experience, they later opted to use headless bioabsorbable screws and/or chondral darts. This was chosen to reduce the risk of screw migration and cartilage wear on the opposing distal tibial articular surface.

### Postoperative Management

Postoperatively, patients were immobilized in a non-weightbearing lower leg cast for 5 to 6 weeks and were provided crutches. At 2 weeks postoperatively, a cast change, wound assessment, and removal of stiches were performed. After the initial 5 to 6 weeks of non-weightbearing casting, the patient was moved to a walking cast for another 5 to 6 weeks with gradual build-up of loading as tolerated up to 100% of body weight. The total 10 weeks of casting was later introduced in the study based on expert opinion and previous experience of the treating surgeons.^6^ This protocol was applied in the last 6 consecutive patients. The previous patients from the start of the study all underwent a total of 12 weeks of casting. All patients underwent a CT scan after 10 or 12 weeks of casting to assess consolidation of the medial malleolus osteotomy and fragment union. Additionally, all patients were referred to a physical therapist for a rehabilitation program. Such a program concerned a phased and personalized rehabilitation protocol. This meant that patients first had to regain range of motion, a normal walking pattern, neuromuscular control, and joint stability. Hereafter, patients progressed to strength and further stability training and to sports- or activity-specific training with the eventual goal to return to a desired level of activity.

### Data Collection

Patient and treatment characteristics at baseline were collected from the hospital electronic patient records. Data extraction was performed by using a predefined extraction format. Baseline patient characteristics included sex, age, body mass index (BMI), smoking status, previous injury circumstances (traumatic or nontraumatic), and previous foot and ankle surgery. Treatment characteristics collected were primary or nonprimary (ie, failed previous surgical treatment) nature of the lesion, surgical approach (medial or lateral), osteotomy use and location (medial malleolar or distal fibular), type of fixation (bioabsorbable screw, chondral dart, or metal screw), and any concomitant procedures at index surgery.

### PRO Measures

PRO measures (PROMs) were collected electronically using Castor in a prospective manner.

The primary outcome of this study was the comparison in numeric rating scale (NRS) of pain during walking between the preoperative score to the 2-year postoperative follow-up score. The NRS of pain is an 11-point Likert scale ranging from 0 (no pain) to 10 (worst imaginable pain). Other PROMs collected were the NRS of pain during rest, during running, and during stairclimbing, as well as the Foot and Ankle Outcome Score (FAOS) and the 36-Item Short Form Health Survey (SF-36). The FAOS is a validated questionnaire for OLT and consists of 42 questions distributed over 5 subscales (Symptoms, Pain, Activities of Daily Living, Sport and Recreation [Sport/Rec], and Quality of Life); it is measured from 0 (lowest) to 100 (highest).^2^ The SF-36 is a general health questionnaire with 2 subscales, the Physical Component Summary (PCS) and Mental Component Summary (MCS).

### Complications and Reoperations

When patients presented at the outpatient clinic during the 2-year study period, the presence of any complications or reoperations was prospectively assessed and recorded in the electronic patient records. Reoperations were divided into revision surgery (ie, reoperation for recurrent OLT) and reoperation of the index foot or ankle for any other reason.

### Radiological Outcome Measures

Baseline CT scans were assessed for all patients by 2 independent measurers (Q.R. and J.D.). Baseline radiological data consisted of the maximal diameter of the lesion and fragment in the anteroposterior direction, mediolateral direction, and depth. The location of the lesion was reported according to a 9-grid scheme.^18^ Additionally, the presence of preoperative cysts, regardless of size, was evaluated.

Follow-up CT scans 1 year after LDFF were assessed for union of the osteochondral fragment by the 2 measurers. CT scans were available for 33 out of 34 patients. The definition of fragment union was modified from Choi et al^19^ and defined as union (75% bony healing), partial union (75%-25% bony healing), or nonunion. The union rate was calculated as union and partial union over the total number of ankles.

### Statistical Analysis

A sample size calculation for the primary outcome, the NRS during walking from preoperatively to 2 years postoperatively, indicated that 26 ankles were needed to detect a difference in means of 1.5 out of 10, assuming a standard deviation of 2.5 using a Wilcoxon signed-rank test with a 2-sided .05 significance level and 80% power (nQuery Advisor 8.5; Statistical Solutions Ltd).^28^ To correct for a potential loss to follow-up of 25%, the required minimum sample size for the present study was 33 cases.

Statistical analysis was performed using Stata 17 (StataCorp LP). A 2-sided significance level of *P* < .05 was considered significant. Baseline characteristics were depicted as means with standard deviations for continuous variables if normally distributed, and frequencies with percentages for dichotomous and categorical outcomes. Data normality was assessed visually with boxplots and histograms. The preoperative and 2-year postoperative PROMs, including the primary outcome, were compared with a Wilcoxon signed-rank test. The change in PROMs was reported as “improvement” due to the different effect directions of the outcome measures. A subanalysis was performed for the association of baseline variables with the change in primary outcome from baseline to 2-year follow-up, for which a Mann-Whitney *U* test was performed for dichotomous variables, Kruskal-Wallis for categorical variables, and Spearman rho test for continuous variables. For the union rate, 95% CIs were calculated using the Wilson score method (without continuity correction). A logistic regression was used to assess the influence of baseline variables on the risk of nonunion. Last, a subanalysis on baseline characteristics of included patients and patients who had an incomplete follow-up or were lost to follow-up was performed.

An interobserver reliability assessment for the lesion size and fragment size measurements was conducted with a 2-way mixed-effects intraclass correlation coefficient (ICC) model with absolute agreement. ICC analysis outcomes were interpreted as 0.41 to 0.60 indicating fair agreement, 0.61 to 0.80 moderate agreement, and 0.81 to 1.00 substantial agreement.^30^ A Cohen kappa analysis was used to assess the reliability of union assessment and the novel descriptive morphological classification. Agreement for the Cohen kappa test was interpreted as substantial if *k* = 0.61 to 0.8 and near-perfect if *k* > 0.81.^12^

## Results

**Figure 1.**
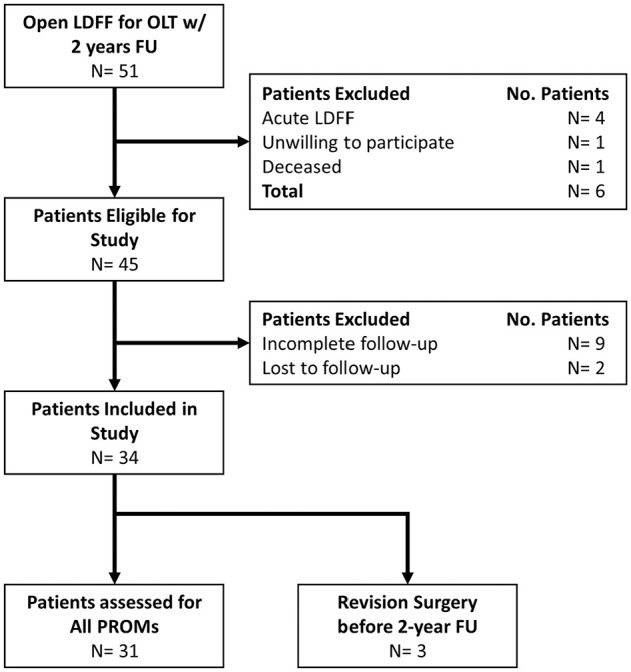
Flowchart of the patient selection process. FU, follow-up; LDFF, lift, drill, fill and fix; PROM, patient-reported outcome measure.

In total, 51 patients were assessed for inclusion, of which 34 patients (34 ankles) were assessed for the primary outcome and/or secondary outcomes (Figure 1). A detailed overview of all patient and treatment characteristics is available in Table 2 and lesion characteristics in Table 3. The subanalysis on baseline characteristics of included patients and patients that had an incomplete follow-up or were lost to follow-up did not show any significant differences (Appendix Table A1).

**Table 2:** Overview of Patient and Treatment Characteristics (N = 34)*^*a*^*

Patient Characteristics	Value	Percentage Reported
Sex, male	18 (53)	100
Age, y	19.3 ± 5.1	100
Smoking status, active smoker	6 (18)	94
BMI, kg/m^2^	24.1 ± 5.5	100
Previous traumatic etiology		100
No previous described trauma	14 (41)	
Inversion/eversion	13 (38)	
Distortion	4 (12)	
Fall from height	2 (6)	
Other	1 (3)	
Previous ankle surgery*^*b*^*		100
Ankles	8 (24)	
Total previous procedures	13	
Detailed, previous surgeries, n = 13		
Previous fixation		
Open	1 (8)	
Arthroscopic	3 (23)	
Arthroscopic BMS		
Other OLT	1 (8)	
OLT treated with LDFF	1 (8)	
Hardware removal		
Open, osteotomy screws	1 (8)	
Arthroscopic removal of 1 fixation screw	1 (8)	
Retrograde drilling	2 (15)	
Clubfoot correction	1 (8)	
Achilles tenotomy	1 (8)	
Excision of os trigonum	1 (8)	
Treatment characteristics		
Approach		100
Medial	20 (59)	
Lateral	14 (41)	
Osteotomy	20 (59)	100
Detailed, osteotomies, n = 20		
Medial malleolar	19 (95)	
Fibular	1 (5)	
Screws per patient, n, mean	1.4	100
Detailed		
Bioscrew	22 (65)	
Metal screw	6 (18)	
Chondral dart	1 (3)	
Combination*^*c*^*	5 (15)	
Bone grafting		100
Distal tibia	28 (82)	
Iliac crest	5 (15)	
None	1 (3)	

a Data are presented as mean ± SD or n (%) unless otherwise indicated. BMI, body mass index; BMS, bone marrow stimulation; LDFF, lift, drill, fill and fix; OLT, osteochondral lesions of the talus.

b A patient could have had >1 previous surgical procedure.

c Combination of bioscrew with chondral dart.

**Table 3:** Overview of Lesion Characteristics (N = 34)

Lesion Characteristics	Value
Primary lesion	28 (82)
Presence cyst	20 (59)
Lesion location	
Zone 1, anteromedial	0 (0)
Zone 2, anterocentral	0 (0)
Zone 3, anterolateral	7 (20)
Zone 4, centromedial	6 (18)
Zone 5, central	1 (3)
Zone 6, centrolateral	3 (9)
Zone 7, posteromedial	15 (44)
Zone 8, posterocentral	0 (0)
Zone 9, posterolateral	2 (6)
Lesion size, mm	
Anteroposterior	18.7 ± 4.3
Mediolateral	12.0 ± 2.4
Depth	8.5 ± 2.5
Lesion area, mm^2^	178.3 ± 57.1
Fragment size, mm	
Anteroposterior	14.4 ± 4.1
Mediolateral	9.4 ± 1.9
Depth	5.2 ± 1.6
Lesion area, mm^2^	108.2 ± 37.9

### PRO Measures

**Figure 2.**
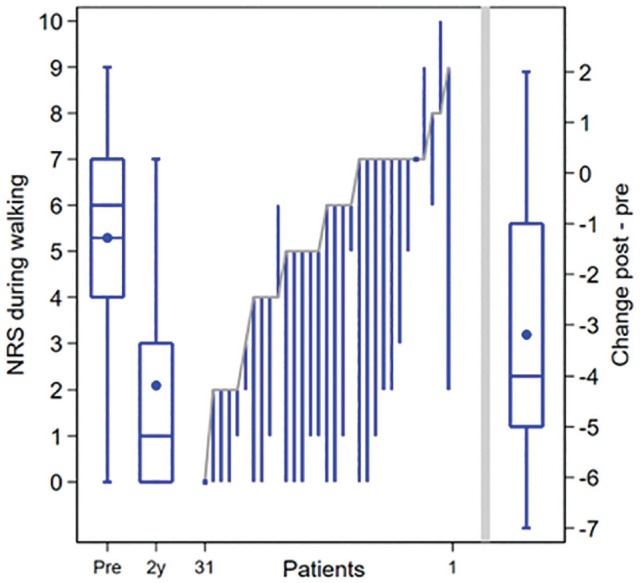
Preoperative (Pre) to 2-year postoperative (Pro) change in numeric rating scale (NRS) during walking, depicted per individual patient (cases 1 - 31). On the left side, boxplots depict the preoperative and 2-year postoperative outcomes on a group level (IQR is indicated by a box with line as median value, dot depicts mean value, and whiskers depict absolute values). On the right, the change on a group level from preoperative to 2 years postoperatively is depicted.

The primary outcome, NRS pain during walking, significantly improved from a median of 6 (IQR, 4-7) out of 10 preoperatively to 1 (IQR, 0-3) out of 10 at final follow-up (*P* < .01) (Figure 2). There was no association between baseline factors and change in primary outcome between baseline and 2-year follow-up (Appendix Table A2). All other PROMs significantly improved compared with the preoperative situation, except for the MCS of the SF-36 (Table 4).

**Table 4:** Secondary Patient-Reported Outcome Measures*^*a*^*

Outcome n = 31	Preoperative	2-Year Follow-up	*P*-Value
NRS pain during			
Rest	3 (1-4)	0 (0-2)	**<.01**
Walking	6 (4-7)	1 (0-3)	**<.01**
Running (n = 29)	8 (7-10)	3 (0-6)	**<.01**
Stair climbing (n = 29)	5 (2-7)	1 (0-3)	**<.01**
FAOS (n = 30)			
Symptoms	50 (39-61)	64 (50-79)	**<.01**
Pain	53 (42-64)	86 (69-97)	**<.01**
ADL	66 (54-85)	96 (88-100)	**<.01**
Sport/Rec	33 (20-45)	73 (55-85)	**<.01**
QOL	38 (31-50)	56 (50-69)	**<.01**
SF-36 (n = 29)			
PCS	37.4 (32.6-41.7)	47.8 (38.1-52.2)	**<.01**
MCS	49.8 (35.7-58.1)	53.3 (46.3-57.8)	.20

a Data are presented as median (IQR). Bold values indicate a significant difference. ADL, Activities of Daily Living; FAOS, Foot and Ankle Outcome Score; MCS, Mental Component Summary; NRS, numeric rating scale; PCS, Physical Component Summary; QOL, Quality of Life; SF-36, 36-Item Short Form Health Survey; Sport/Rec, Sport and Recreation.

### Radiological Outcomes

**Figure 3.**
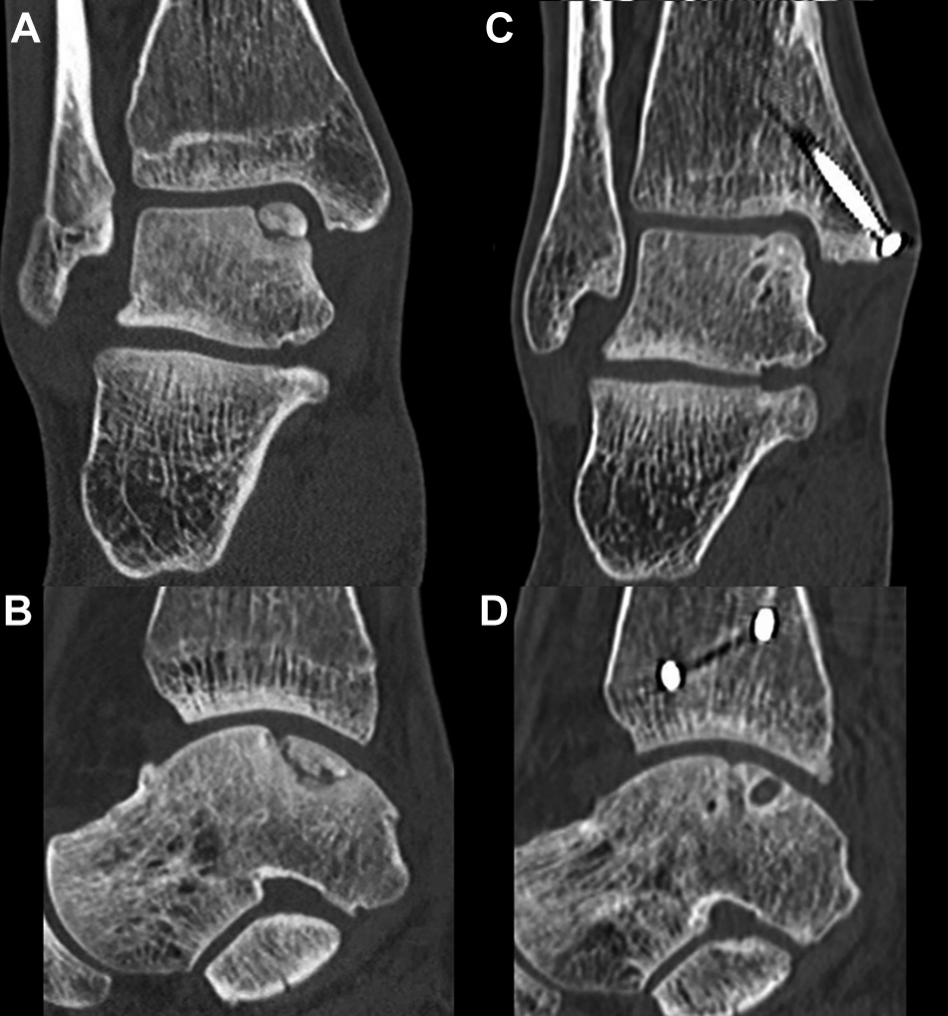
Preoperative coronal (A) and sagittal (B) and 2-year postoperative (C, D) computed tomography images of an 18-year-old male with a posteromedial osteochondral fragment of the talus, who underwent open lift, drill, fill, and fix with 1 bioscrew. The postoperative radiolucency on the talus is the bioscrew in situ.

**Figure 4.**
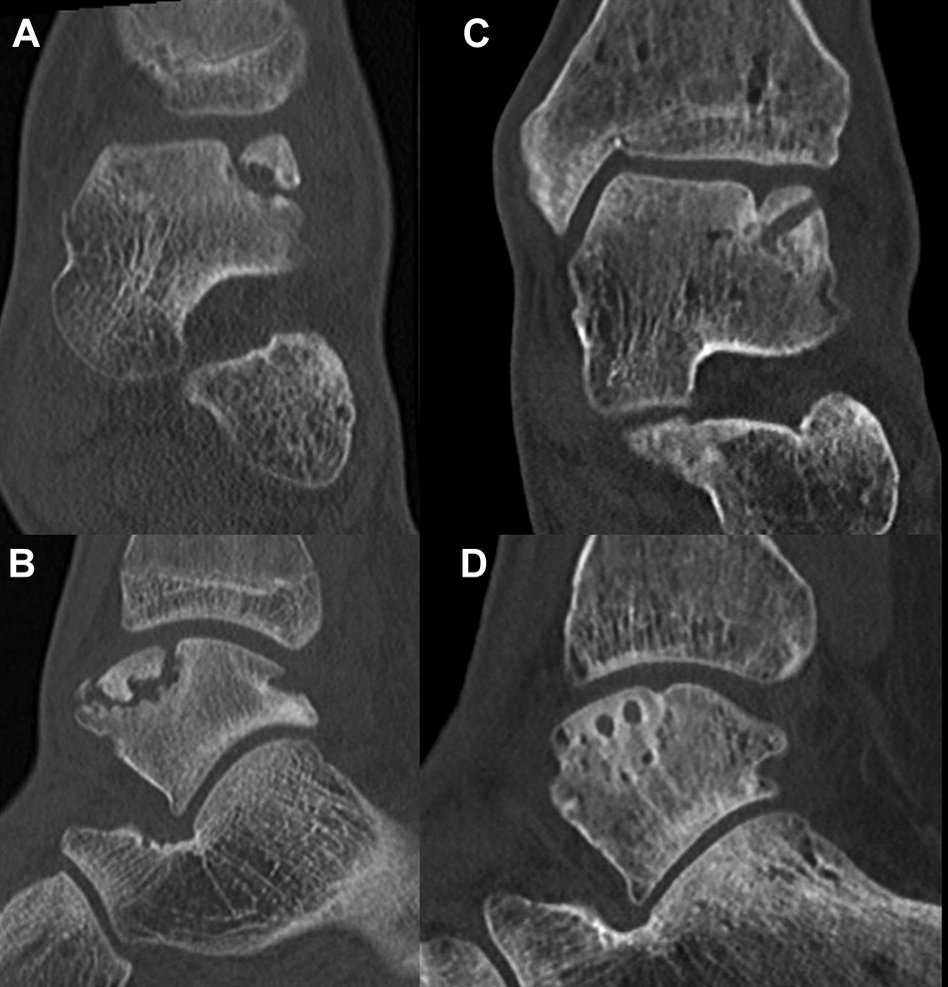
Preoperative coronal (A) and sagittal (B) and 1-year postoperative (C, D) computed tomography images of a 16-year-old male with an anterolateral osteochondral fragment of the talus, who underwent open lift, drill, fill, and fix with 2 bioscrews. The postoperative radiolucencies on the talus are the bioscrews in situ.

On 1-year follow-up CT scans, a union rate of 91% (95% CI, 76-98) was observed. Patients who showed union had full union in 27 (90%) cases and partial union in 3 (10%). Two case examples are shown in Figures 3 and 4. BMI of ≥30 kg/m^2^ was significantly associated with fragment nonunion (odds ratio [OR], 1.39; 95% CI, 1.04-1.84; *P* = .02), and all cases of nonunion had a BMI >30. The fragment area (OR, 0.99; 95% CI, 0.98-1.02; *P* = .90) or depth (OR, 0.90; 95% CI, 0.41-1.96; *P* = .80) was not associated with union (Appendix Table A3). The osteotomy healed uneventfully in 100% of the cases. The outcomes of the interobserver analysis for the measurement of lesion size are available in Appendix Table A4.

### Complications, Revision Surgery, and Reoperations

In this cohort, 2 (6%) complications were observed. Of these, 1 patient had delayed superficial wound healing without signs of infection that required an additional cast change at 4 weeks postoperatively and thereafter resolved uneventfully. Additionally, 1 patient developed a complex regional pain syndrome postoperatively, for which a multidisciplinary pain rehabilitation treatment was commenced.

When considering revision surgery, 3 (9%) patients underwent revision surgery at a mean of 13.7 (range, 10-16) months follow-up, all due to a symptomatic nonunion of the fragment. Of these 3 patients, 1 patient underwent arthroscopic bone marrow stimulation (BMS), and 2 patients underwent Talar OsteoPeriostic grafting from the Iliac Crest.^3^ All achieved adequate results and did not require additional surgery afterward. A detailed overview of the patient characteristics for revision cases is presented in Appendix Table A5. In addition to the revision surgeries, a total of 9 patients underwent additional surgery after the LDFF procedure within the study period, which was osteotomy hardware removal in 6 out of 9 patients (Appendix Table A6). None required screw removal used in fixation of the lesion.

## Discussion

The main finding of this study is that open LDFF for chronic OLT with an osteochondral fragment resulted in favorable patient-reported outcomes and pain reduction at 2-year follow-up. Moreover, 91% of patients showed union of the fragment at 1-year CT evaluation, and it was found that BMI was associated with a significantly increased risk of nonunion. The revision rate was 9% and reoperation rate 24% (majority symptomatic hardware removal). These findings show that open LDFF is a clinically effective treatment method for chronic OLT with an osteochondral fragment amenable for fixation. Moreover, these findings may allow surgeons for a wider range of treatment options for specific lesion types.

The indication for fixation is debated. A 2019 consensus statement from experts in the field stated that osteochondral lesions with an osteochondral fragment require a bony stock of ≥10 mm in diameter and ≥3 mm depth to be eligible for fixation.^21^ However, recent work by Nakasa et al^15^ has shown that even in smaller (<100 mm^2^) lesions fixation yields good clinical and union outcomes. The indication for fixation of OLT, therefore, seems to be expanding.

A recent systematic review and meta-analysis, including 241 ankles among 10 studies, observed a pooled treatment success rate of 91%, which was based on cutoff values for commonly used patient- or physician-reported outcome measures.^23^ From these studies, 88% of cases reported outcomes of fixation through an open approach and 44% of cases reported the use of screw or pin fixation.^23^ From the present literature, Nakasa et al^15^ reported the largest case series of 36 ankles fixed with poly-L-lactide pins. The authors reported an improvement of the American Orthopaedic Foot & Ankle Society score from 71.1 (SD, 2.6) preoperatively to 97.2 (SD, 4.3) at a mean 23 months postoperatively. Moreover, the aforementioned study found postoperative clinical scores of patients that underwent fixation to be statistically significantly higher compared to arthroscopic BMS. Haraguchi et al^5^ reported the largest cohort of patients fixed using a bone peg in 45 ankles and reported an improvement of the Japanese Society for Surgery of the Foot ankle/hindfoot scale from 63.5 (SD, 17.9) preoperatively to 93.0 (SD, 6.6) at a mean 43.2 months postoperatively. Moreover, the outcomes observed in the present study concur with these improvements in clinical outcomes and, similarly, with previous findings of the arthroscopic LDFF procedure.^11^ The present study found no baseline factors to be associated with the improvement of pain outcomes during walking (primary outcome). Similarly, no baseline factors were found to correlate with postoperative PROMs in previous studies on fixation for OLT.^5,9,15,19^ This may be due to underpowering in these studies for such an analysis, as well as the current study, and should be investigated in larger cohorts.

Although sports outcomes were not formally assessed in the present study, pain scores during running and the FAOS Sport subscale showed a statistically and clinically relevant improvement, albeit with residual pain during running and a moderate to fair improvement in the FAOS Sport score. These outcomes may suggest that fixation could, similarly, result in good sport outcomes. Nakasa et al^15^ reported that, in their previously mentioned series of 36 patients that the ankle activity scale remained stable from 6.5 (SD, 2.2) preoperatively to 6.3 (SD, 2.2) at a mean of 23 months follow-up, and it was found to be superior compared to BMS. Schuh et al^29^ reported in their retrospective evaluation of 20 ankles treated with K-wire fixation of the osteochondral fragment that all patients returned to their preoperative sports and work, but did not define return to sports per level or specific sport(s). In the cases included in the present study, it was the clinical experience of the authors that the main goal for patients to undergo surgical intervention was either pain reduction during activities of daily living, such as walking, or pain reduction during sports or work. Additionally, the use of the 10- to 12-week immobilization period could affect the sport outcomes due to prolonged immobilization. Current trends are to decrease the immobilization period for open OLT surgery, as it shows safe outcomes.^6,7^ In the fixation literature, no consensus is reached on the appropriate immobilization period for OLT fixation healing, with nonweightbearing periods of 4 to 6 weeks being reported, and subsequent partial weightbearing either protected or unprotected.^1,5,8,10,17,19,21^ The authors acknowledge that during the course of this study, the immobilization period was shortened and no adverse outcomes were noted as this could have influenced outcomes, and to date commonly using a walker boot during weightbearing immobilization in compliant patients. Consensus should be reached on the adequate immobilization period for fixation of OLT.

### Radiological Outcomes

The fragment union rate observed in the present study was 91%. This concurs with the literature, where the aforementioned systematic review on fixation observed union in 91% of ankles.^23^ The present study found that a BMI ≥30 kg/m^2^was associated with an increased risk for nonunion. We hypothesize that obesity may result in increased peak forces during early weightbearing, thereby increasing the risk of (subtle) fragment instability and inadequate bone healing, leading to a delayed union or nonunion of the fragment. Clinically, these findings can be considered when counseling patients for surgery. Moreover, in the elective setting, an aim toward optimal weight reduction preoperatively for obese (BMI ≥ 30) patients may be considered or an extension of the postoperative partial weightbearing period of the ankle (for example with a walking boot). The authors do warrant caution when interpreting the findings of this analysis due to the relatively low number of patients in the present cohort along with statistical fragility. Further studies in large cohorts should assess the effect of BMI and other patient factors on union outcomes for fixation of OLT. Additionally, it was observed in the present study that no osteotomy nonunions occurred. This concurs with the literature, where similarly, no osteotomy nonunions have been reported in fixation cases.^23^ Although the aim should be to approach the lesion in the most minimally invasive method possible, a concomitant osteotomy during fixation of OLT seems to be safe and is therefore justified in case the access to the lesion is otherwise limited. Moreover, further research will have to elucidate whether the open approach results in similar outcomes to an arthroscopic approach for fixation of OLT and what the effect of osteotomy use is on patient outcomes.

### Complications, Revision Surgery, and Reoperations

In the present study, 2 complications were recorded. Of these, 1 case had persistent complaints of complex regional pain syndrome requiring further treatment, a complication that is seen in up to 4% of foot and ankle surgery cases.^22^

The revision rate for fixation reported in the literature is 6%.^23^ Among these, Rak Choi et al^19^ and Kramer et al^9^ both reported 5 revision cases in 26 and 18 ankles, respectively. Rak Choi et al reported that all had a symptomatic nonunion as the reason for revision surgery, which was conducted through removal of the fragment and bone grafting from the lateral calcaneus. This concurs with the findings in this study, where it was observed that up to 2-years follow-up all 3 patients underwent revision for nonunion. Kramer et al did not report the reason for revision, however. Although not observed in the present cohort, there is a mismatch in the literature^23^ regarding the revision rate (6%) and nonunion rate (9%), which suggests some patients may have a nonsymptomatic nonunion. With caution, it could be stated that there are patients who do not achieve union but do not have clinically relevant complaints. As such, it is critical to thoroughly evaluate a patient with a nonunion after fragment fixation and to consider watchful neglect with radiological follow-up in case of a nonsymptomatic case.

The reoperation rate in the present study (24%) was higher compared with the literature (9%)^23^ and arthroscopic LDFF (0%).^11^ We hypothesize that this is largely due to the symptomatic hardware removal after osteotomy of 67% in this cohort. This is supported by the 72% hardware removal rate reported by Meisterhans et al^13^ in their series of 67 ankles who underwent open surgery for an OLT. The present literature on fixation shows an osteotomy rate of 45%,^23^ compared with the 59% reported in this study, which could be a contributing factor to the reoperation rate observed in this study. Our experience shows that most medially located lesions require an osteotomy to gain access to the joint while most lateral lesions did not. As previously discussed, access to the lesion is paramount in optimal screw placement during fixation, thus requiring an osteotomy. As such, the authors believe that it is important to counsel patients on these findings and to only remove hardware in case of complaints. Future studies may further compare the morbidity and success of medially versus laterally fixed OLT, where factors such as the necessity to remove hardware may play a role in outcomes.

### Strengths and Limitations

The strengths of this study are its prospective design, the inclusion of a prospective sample size calculation for the primary outcome, and two measurers for the radiological data collection with a moderate to excellent interobserver reliability.

However, the present study is not without limitations. First, this study concerns a noncomparative case series. Second, 24% of patients had an incomplete follow-up or were lost to follow-up. From these, the majority were patients who failed to complete a baseline questionnaire. Furthermore, no significant differences in baseline characteristics among excluded and included patients were observed, suggesting that their exclusion may not have changed the primary findings of the study. Third, the authors warrant caution in the interpretation of the subanalyses performed in this study, as these may be underpowered. Fourth, 1 patient was not included in the prospective CT evaluation at 1-year follow-up, as the patient did not undergo the examination, which could have had an effect on the union rate and its subanalyses.

## Conclusion

Open LDFF results in a favorable pain reduction and good clinical outcomes for chronic OLT up to 2-year follow-up. The procedure achieves a 91% fragment union rate, while patients with obesity may be at a higher risk of fragment nonunion. The revision rate was 9% and reoperation rate 24% (majority symptomatic hardware removal). These findings show that open LDFF is a clinically effective treatment method for chronic OLT with an osteochondral fragment amenable for fixation.

## Authors

Q.G.H. Rikken, MD (Department of Orthopedic Surgery and Sports Medicine, Amsterdam UMC, Location AMC, University of Amsterdam, Amsterdam, the Netherlands; Amsterdam Movement Sciences, Programs Sports and Musculoskeletal Health, Amsterdam, the Netherlands; Academic Center for Evidence-based Sports medicine, Amsterdam UMC, Amsterdam, the Netherlands; Amsterdam Collaboration on Health & Safety in Sports, International Olympic Committee Research Center, Amsterdam UMC, Amsterdam, the Netherlands); Jari Dahmen, MD, BSc (Department of Orthopedic Surgery and Sports Medicine, Amsterdam UMC, Location AMC, University of Amsterdam, Amsterdam, the Netherlands; Amsterdam Movement Sciences, Programs Sports and Musculoskeletal Health, Amsterdam, the Netherlands; Academic Center for Evidence-based Sports medicine, Amsterdam UMC, Amsterdam, the Netherlands; Amsterdam Collaboration on Health & Safety in Sports, International Olympic Committee Research Center, Amsterdam UMC, Amsterdam, the Netherlands); Kaj T.A. Lambers, MD, PhD (Amsterdam Movement Sciences, Programs Sports and Musculoskeletal Health, Amsterdam, the Netherlands; Academic Center for Evidence-based Sports medicine, Amsterdam UMC, Amsterdam, the Netherlands; Amsterdam Collaboration on Health & Safety in Sports, International Olympic Committee Research Center, Amsterdam UMC, Amsterdam, the Netherlands; Reinier Haga Orthopaedic Centre, Zoetermeer, the Netherlands); Kaj S. Emanuel, MSc, PhD (Amsterdam Movement Sciences, Programs Sports and Musculoskeletal Health, Amsterdam, the Netherlands; Academic Center for Evidence-based Sports medicine, Amsterdam UMC, Amsterdam, the Netherlands; Amsterdam Collaboration on Health & Safety in Sports, International Olympic Committee Research Center, Amsterdam UMC, Amsterdam, the Netherlands); Sjoerd A.S. Stufkens, MD, PhD (Department of Orthopedic Surgery and Sports Medicine, Amsterdam UMC, Location AMC, University of Amsterdam, Amsterdam, the Netherlands; Amsterdam Movement Sciences, Programs Sports and Musculoskeletal Health, Amsterdam, the Netherlands; Academic Center for Evidence-based Sports medicine, Amsterdam UMC, Amsterdam, the Netherlands; Amsterdam Collaboration on Health & Safety in Sports, International Olympic Committee Research Center, Amsterdam UMC, Amsterdam, the Netherlands); Gino M.M.J. Kerkhoffs, MD, PhD (Department of Orthopedic Surgery and Sports Medicine, Amsterdam UMC, Location AMC, University of Amsterdam, Amsterdam, the Netherlands; Amsterdam Movement Sciences, Programs Sports and Musculoskeletal Health, Amsterdam, the Netherlands; Academic Center for Evidence-based Sports medicine, Amsterdam UMC, Amsterdam, the Netherlands; Amsterdam Collaboration on Health & Safety in Sports, International Olympic Committee Research Center, Amsterdam UMC, Amsterdam, the Netherlands); Amsterdam Ankle Cartilage Team: J. Nienke Altink, MD (Department of Orthopaedic Surgery and Sports Medicine, Amsterdam UMC, Location AMC, University of Amsterdam, Amsterdam, the Netherlands; Amsterdam Movement Sciences, Programs Sports and Musculoskeletal Health, Amsterdam, the Netherlands; Academic Center for Evidence-based Sports medicine, Amsterdam UMC, Amsterdam, the Netherlands); Christiaan J.A. van Bergen, MD, PhD (Amphia Hospital, Breda, the Netherlands); Peter A.J. de Leeuw, MD, PhD (Flevoziekenhuis, Almere, the Netherlands); Rover Krips, MD, PhD (Flevoziekenhuis, Almere, the Netherlands); and Mikel L. Reilingh, MD, PhD (St. Antonius Hospital, Utrecht, the Netherlands).

## Appendix

**Appendix Table A1:** Comparison of Baseline Characteristics of Included Patients Versus Patients with Incomplete- or Lost-to-follow-up*^*a*^*

	Total Patients Included (N = 34)	Incomplete or Lost to Follow-up (n = 11)	*P-Value*
Patient characteristics			
Sex, male	18 (53)	8 (73)	N.S.
Age, y	19.3 ± 5.1	17.3 ± 2.7	N.S.
Smoking status, active smoker	6 (18)	0 (0)	N.S.
BMI	24.1 ± 5.5	22.5 ± 2.9	N.S.
Traumatic etiology			N.S.
No trauma	14 (41)	6 (55)	
Inversion/eversion	13 (38)	4 (36)	
Distortion	4 (12)	0 (0)	
Fall from height	2 (6)	0 (0)	
Other	1 (3)	1 (9)	
Treatment characteristics			
Approach			N.S.
Medial	20 (59)	6 (55)	
Lateral	14 (41)	5 (45)	
Osteotomy	20 (59)	5 (45)	N.S.
Medial malleolar	19 (95)	5 (100)	N.S.
Fibular	1 (5)	0 (0)	
Number of screws, mean	1.4	1.4	N.S.
Type of screw, n (%)			N.S.
Bioscrew	22 (65)	8 (73)	
Metal screw	6 (17)	3 (27)	
Chondral dart	1 (3)	0 (0)	
Combination^*b*^	5 (15)	0 (0)	
Lesion characteristics			
Primary lesion	28 (82)	9 (82)	N.S.
Lesion location			N.S.
Zone 1	0 (0)	0 (0)	
Zone 2	0 (0)	0 (0)	
Zone 3	7 (20)	2 (18)	
Zone 4	6 (18)	1 (9)	
Zone 5	1 (3)	0 (0)	
Zone 6	3 (9)	3 (27)	
Zone 7	15 (44)	5 (46)	
Zone 8	0 (0)	0 (0)	
Zone 9	2 (6)	0 (0)	
Lesion size, mm			
Anteroposterior	18.7 ± 4.3	20.5 ± 5.1	N.S.
Mediolateral	12.0 ± 2.4	12.5 ± 2.7	N.S.
Depth	8.5 ± 2.5	9.1 ± 3.2	N.S.
Fragment size, mm			
Anteroposterior	14.4 ± 4.1	16.7 ± 5.0	N.S.
Mediolateral	9.4 ± 1.9	9.5 ± 1.9	N.S.
Depth	5.2 ± 1.6	6.5 ± 2.1	N.S.

a Data are presented as mean ± SD or n (%) unless otherwise indicated. BMI, body mass index; N.S., nonsignificant.

b Combination of bioscrew and chondral dart.

**Appendix Table A2:** Subanalysis of Baseline Factors on Primary Outcome*^a^*

Variable	Median change in NRS during weightbearing from baseline to 2-year follow-up	*P-Value*
Dichotomous variables		
Sex		.50
Male (n = 17)	5.0 (1-5)	
Female (n = 14)	3.5 (2-4)	
Smoking		.40
Yes (n = 5)	5 (1-6)	
No (n = 26)	3.5 (1.5-5)	
Osteotomy		.30
Yes (n = 18)	4 (1-5)	
No (n = 13)	4 (2-6)	
Cyst		.10
Yes (n = 17)	5 (2-5)	
No (n = 14)	2 (1-4)	
Lesion location*^*b*^*		.20
Medial (n = 19)	4 (1-5)	
Lateral (n= 12)	5 (2-6)	
>2 groups		
Fixation device		.90
Bioscrew (n = 21)	4 (2-5)	
Metal screw (n = 6)	3.5 (0-6)	
Chondral dart (n = 3)	4 (0-7)	
Combination (n = 1)	2 (N/A)	
Continuous variables	Correlation coefficient	
Age	0.03	.90
Body mass index	0.01	.90
Lesion size		
Lesion area, mm^2^	0.01	.90
Depth	–0.01	.60
Fragment size		
Lesion area, mm^2^	–0.11	.60
Depth	0.05	.90

a Number of patients may vary from baseline data, as 3 failure cases were not included in this analysis. N/A, not available.

b Medial was defined as Raikin et al zones 1, 4, or 7. Lateral was defined as zones 3, 6, or 9.

**Appendix Table A3:** Association of Baseline Factors With Nonunion^*a*^

	OR (95% CI)	*P*
Fragment area	0.99 (0.98-1.02)	.90 (N.S.)
Fragment depth	0.90 (0.41-1.96)	.80 (N.S.)
Presence of cysts		.2 (N.S.)
Location, medial or lateral		.7 (N.S.)
BMI	1.39 (1.05-1.84)	**.02**

a BMI, body mass index; N.S., nonsignificant; OR, odds ratio.

**Appendix Table A4:** Interobserver Reliability Measurements for Radiological Parameters*^*a*^*

	Interobserver Reliability
ICC lesion	
AP	0.95 (0.75-0.97)
ML	0.71 (0.13-0.90)
Depth	0.91 (0.60-0.94)
ICC fragment	
AP	0.98 (0.92-0.99)
ML	0.65 (0.01-0.88)
Depth	0.95 (0.86-0.98)
Cohen’s kappa union	0.77 (standard error: 0.22)

AP, anteroposterior; ICC, intraclass correlation coefficient; ML, mediolateral.

**Appendix Table A5:** Baseline Demographics and Treatment Characteristics for Failure Cases^*a*^

	Patient Characteristics				
Case	Sex	Age, y	BMI, kg/m^2^	Injury Etiology	Previous Ankle Surgery
1	F	18	31.0	Nontraumatic	No
2	M	15	32.3	Traumatic	No
3	F	15	41.8	Nontraumatic	No
	Lesion Characteristics				
Case	Lesion Location	Fragment Size, mm		Morphology	
1	Zone 7	AP: 14 ML: 10 Depth: 3		Undisplaced, multifragmentary	
2	Zone 6	AP: 16 ML: 11 Depth: 6		Undisplaced, single fragment	
3	Zone 7	AP: 17 ML: 7 Depth: 6		Undisplaced, single fragment	
	Treatment and Revision Characteristics				
Case	Screw Type	Reason Revision Surgery	Revision Procedure		
1	1 bioscrew	Symptomatic nonunion fragment	Arthroscopic debridement and BMS		
2	1 bioscrew +1 chondral dart	Symptomatic nonunion fragment	TOPIC		
3	1 bioscrew +1 chondral dart	Symptomatic nonunion fragment	TOPIC		

a AP, anteroposterior; BMI, body mass index; BMS, bone marrow stimulation; F, female; M, male; ML, mediolateral; TOPIC, Talar OsteoPeriostic grafting from the Iliac Crest.

**Appendix Table A6:** Subsequent Surgeries After LDFF Procedure (n = 9)

Indication and Procedure	No. of Patients (% of total)
Irritation of osteotomy screws	
Hardware removal	5 (56)
Anterior bony impingement	
Arthroscopic removal of anterior osteophytes	1 (11)
Posterior bony impingement due to traumatized os trigonum	
Posterior ankle arthroscopy with removal of os trigonum	1 (11)
Irritation of osteotomy screws and peroneal tendon tear	
Hardware removal and personal tendon suturing	1 (11)
Symptomatic syndesmotic instability tightrope syndesmotic stabilization,	
elective removal of hardware, second-look anterior ankle arthroscopy with removal of loose body	1 (11)
